# The Role of Bile Acid Excretion in Atherosclerotic Coronary Artery Disease

**DOI:** 10.1155/2012/949672

**Published:** 2011-09-12

**Authors:** Gideon Charach, Alexander Rabinovich, Ori Argov, Moshe Weintraub, Pavel Rabinovich

**Affiliations:** Department of Internal Medicine “C”, Tel Aviv Sourasky Medical Center, Tel Aviv 64239, Israel

## Abstract

The impact of cholesterol and different classes of lipoproteins on the development of coronary artery disease (CAD) has been investigated in extensively during the past 50 years. The cholesterol metabolism is dependent on numerous factors, including dietary fat, fractional absorption of dietary cholesterol, tissue stores of cholesterol, endogenous cholesterol synthesis, and fecal bile excretion. Several studies showed significantly lower amounts of bile acid secretion in adult patients with CAD compared to non-CAD patients. Could it be that the inability to efficiently excrete bile acids may lead to CAD development?

## 1. Introduction

While a great deal of attention has been given to the factors that determine cholesterol homeostasis, cholesterol excretion via bile in patients with CAD has not been thoroughly examined [1–3]. 

Clinically, it became obvious that, despite effective cholesterol-modulating treatment (e.g., statins), the development of atherosclerosis cannot be stopped in a significant number of patients. It is known that cholesterol is mainly eliminated from the body via the liver in the form of bile acids [2, 4, 5]. Therefore, in addition to statins, low-density lipoprotein cholesterol (LDL-c) can be reduced by increasing the fecal bile acid waste and by compensatory hepatic upregulation of bile acid synthesis [2, 4]. 

It is reasonable to speculate that a reduced ability to convert cholesterol to bile acids would lead to body cholesterol overload, with the subsequent development of atherosclerosis [6–10]. The aim of this paper is to emphasize the effect of bile acid disposal on coronary artery atherosclerosis. 

## 2. Bile Acid Excretion and Its Relationship to Coronary Atherosclerosis

### 2.1. Animal Studies

Studies in animals have revealed that rodents do not develop experimental atherosclerosis, despite ingestion of a cholesterol-rich diet [2, 11–13]. They were able to react to the overload of cholesterol intake by excreting large amounts of bile acids. The same results were obtained in a study on New Zealand white rabbits and primates which were also fed a cholesterol-rich diet [14]. Animals that excreted large amounts of cholesterol did not develop hypercholesterolemia, whereas those with a less efficient excretion had increased plasma level of cholesterol [14]. Furthermore, the degree of hypercholesterolemia was inversely correlated to the rate of bile acid elimination. These animal experiments suggest that the atherogenic effect of the cholesterol-rich diet closely depends on the animal's ability to eliminate cholesterol in the form of bile acids [12, 13]. It is therefore reasonable to speculate that reduced ability to convert cholesterol to bile acids would lead to cholesterol overload, with the possibility of subsequent enhanced development of atherosclerosis [6–10]. A similar correlation between the elimination of cholesterol in bile and the development of atherosclerosis was suggested for humans.

### 2.2. Human Studies

In an earlier investigation the elimination of bile acid in the feces of patients who had ischemic heart disease was compared with that of healthy controls on the same diet [15]. It was reported that the patients excreted much fewer bile acids than the controls [15].

 Several new studies had shown an inverse relationship between CAD and bile acid excretion [6–10, 16, 17]. There are few human studies dealing with disturbed metabolism of plant sterols (which reflects cholesterol) in postmenopausal CAD women who showed disturbed synthesis and disturbed secretion of bile acids [15, 16]. 

These findings support the hypothesis that CAD patients produce fewer bile acids than individuals without CAD and that reduced production of bile acids could lead to advanced atherosclerosis. These findings are in line with those of the several human studies that showed increased fecal excretion of bile acids to have protective effect on CAD development [6–10, 15–17]. Most of those investigations were done on selected populations using the method described by Grundy et al. [18] for determination of total bile acids. For example, Simonen and Miettinen [19] showed that males with heterozygous familial hypercholesterolemia (FH) and CAD excreted less bile acids than control males with FH and normal coronaries. Rajaratnam et al. showed that postmenopausal women with CAD had inefficient fecal elimination of cholesterol [17].

We recently published a study on a general adult population with and without CAD and found that CAD patients eliminated subnormal amounts of fecal bile acids [3]. 

CAD patients excreted 358 ± 156 mg of total bile acids in comparison to healthy patients 617 ± 293 mg; *P* < 0.01. The differences in excretion were mainly due to lower excretion of deoxycholic (188.29 ± 98.12 mg versus 325.96 ± 198.57 mg; *P* < 0.0001) and less lithocholic acid (115.43 ± 71.89 mg versus 197.27 ± 126.87 mg; *P* < 0.01). Findings of this study based on 36 CAD patients and 37 non-CAD patients with a follow-up period of up to 13 years supported earlier ones and allowed for the reaching of more firm conclusions on the role of the elimination of fecal bile acids in CAD development [3].

## 3. Relationship between Plasma Triglycerides, HDL-Cholesterol, and Bile Acids

HDL-c has been proposed to serve as preferential precursor for bile acid biosynthesis in the liver. Furthermore, a negative relationship between plasma levels of HDL cholesterol and biliary saturation with cholesterol has been reported in healthy females [20].

 In contrast to total cholesterol, LDL-c and HDL-c, there was a correlation between plasma triglycerides and bile acid excretion, but only in the non-CAD group [3]. This can be explained by a rapid and more complete intestinal absorption of triglycerides due to an excess of bile acids which are necessary for the emulsification of fats. CAD patients did not exhibit this effect because the amount of excreted bile acids was significantly lower [3].

## 4. Stroke and Bile Acids Excretion

Atherosclerosis is a disease characterized by lipid accumulation in the vascular wall leading to myocardial infarction or stroke [20–22]. In spite of proven efficacy of the existing drugs, like statins, cardiovascular diseases still remain the most important causes of morbidity and mortality in industrialized countries. A cholesterol-lowering effect can be achieved by reducing cholesterol synthesis or by increasing fecal excretion of bile acids (ileal sodium-dependent bile acid transporter inhibitors). It is important to emphasize that the ability to excrete large amounts of bile acids not only prevents CAD development but also may also prevent atherosclerosis in the cerebral arteries as well [3, 21, 22].

The results of the recent study after a long followup pointed out to a 6-fold higher incidence of ischemic stroke among the CAD patients compared to the non-CAD patients, 7 patients (19%) versus 1 patient (2.7%), and three-fold greater mortality in the CAD group, 9 patients (25%) versus 3 patient (8%) [3]. 

## 5. 7-*α* Hydroxylase: The Main Enzyme Responsible for Bile Acids Excretion

The 7-*α* hydroxylase is the key enzyme in the conversion of cholesterol to bile acids [12, 13, 21, 23, 24]. It mediates the elimination of cholesterol from the plasma and the intracellular compartment, and it facilitates the excretion of bile acids. Thus, enhanced excretion of bile acids in non-CAD individuals can possibly be explained mainly by increased activity and concentration of 7-*α*-hydroxylase [12, 13, 21, 23, 24]. In contrast to it, CAD patients are unable to effectively increase the activity and concentration of 7-*α*-hydroxylase [23]. 

## 6. Future Perspective: Combined Treatment with Statins and Bile Acid Sequestrants

A significant percentage of patients develop atherosclerosis despite statin treatment and suppressed levels of cholesterol. We reason that, by decreasing cholesterol synthesis and increasing the utility of cholesterol, an additive antiatherosclerosis effect might be achieved. This is particularly true in the case of patients at high risk for CAD. To date it is established that these patients require aggressive lipid-lowering therapy. By combining a statin with drugs affecting bile acid and cholesterol absorption an optimal management of dyslipidemia may potentially be ensured. Additional studies are necessary to validate our contention.

## 7. Conclusion

Reviewing of several human studies revealed significantly lower excretion of bile acid in adult patients with CAD compared to non-CAD individuals. The diminished excretion of bile acids might be an independent risk factor for CAD and a potential target for cholesterol lowering treatment.

## References

[1] Lin DS, Connor WE (1980). The long term effects of dietary cholesterol upon the plasma lipids, lipoproteins, cholesterol absorption, and the sterol balance in man: the demonstration of feedback inhibition of cholesterol biosynthesis and increased bile acid excretion. *Journal of Lipid Research*.

[2] Bhat BG, Rapp SR, Beaudry JA (2003). Inhibition of ileal bile acid transport and reduced atherosclerosis in apoE-/- mice by SC-435. *Journal of Lipid Research*.

[3] Charach G, Grosskopf I, Rabinovich A, Shochat M, Weintraub M, Rabinovich P (2011). The association of bile acid excretion and atherosclerotic coronary artery disease. *Therapeutic Advances in Gastroenterology*.

[4] Izzat NN, Deshazer ME, Loose-Mitchell DS (2000). New molecular targets for cholesterol-lowering therapy. *Journal of Pharmacology and Experimental Therapeutics*.

[5] Batta AK, Salen G, Rapole KR (1999). Highly simplified method for gas-liquid chromatographic quantitation of bile acids and sterols in human stool. *Journal of Lipid Research*.

[6] Rajaratnam RA, Gylling H, Miettinen TA (1999). Serum squalene in postmenopausal women without and with coronary artery disease. *Atherosclerosis*.

[7] Rajaratnam RA, Gylling H, Miettinen TA (2000). Independent association of serum squalene and noncholesterol sterols with coronary artery disease in postmenopausal women. *Journal of the American College of Cardiology*.

[8] Glueck CJ, Speirs J, Tracy T, Streicher P, Illig E, Vandegrift J (1991). Relationships of serum plant sterols (phytosterols) and cholesterol in 595 hypercholesterolemic subjects, and familial aggregation of phytosterols, cholesterol, and premature coronary heart disease in hyperphytosterolemic probands and their first-degree relatives. *Metabolism*.

[9] Sudhop T, Gottwald BM, Von Bergmann K (2002). Serum plant sterols as a potential risk factor for coronary heart disease. *Metabolism*.

[10] Assmann G, Cullen P, Erbey J, Ramey DR, Kannenberg F, Schulte H (2006). Plasma sitosterol elevations are associated with an increased incidence of coronary events in men: results of a nested case—control analysis of the Prospective Cardiovascular Munster (PROCAM) study. *Nutrition, Metabolism and Cardiovascular Diseases*.

[11] Post SM, de Crom R, van Haperen R, Van Tol A, Princen HMG (2003). Increased fecal bile acid excretion in transgenic mice with elevated expression of human phospholipid transfer protein. *Arteriosclerosis, Thrombosis, and Vascular Biology*.

[12] Lutton C (1990). Cholesterol and bile acid dynamics: comparative aspects. *Reproduction Nutrition Development*.

[13] Li H, Xu G, Shang Q (2004). Inhibition of ileal bile acid transport lowers plasma cholesterol levels by inactivating hepatic farnesoid X receptor and stimulating cholesterol 7*α*-hydroxylase. *Metabolism*.

[14] Lofland HB, Clarkson TB, St Clair RW, Lehner NDM (1972). Studies on the regulation of plasma cholesterol levels in squirrel monkeys of two genotypes. *Journal of Lipid Research*.

[15] Charach G, Rabinovich PD, Konikoff FM, Grosskopf I, Weintraub MS, Gilat T (1998). Decreased fecal bile acid output in patients with coronary atherosclerosis. *Journal of Medicine*.

[16] Gylling H, Hallikainen M, Rajaratnam RA, Simonen P, Pihlajamäki J, Laakso M (2009). The metabolism of plant sterols is disturbed in postmenopausal women with coronary artery disease. *Metabolism*.

[17] Rajaratnam RA, Gylling H, Miettinen TA (2001). Cholesterol absorption, synthesis, and fecal output in postmenopausal women with and without coronary artery disease. *Arteriosclerosis, Thrombosis, and Vascular Biology*.

[18] Grundy SM, Ahrens EH, Miettinen TA (1965). Quantitative isolation and gas liquid chromatographic analysis of total bile acids. *Journal of Lipid Research*.

[19] Simonen H, Miettinen TA (1987). Coronary artery disease and bile acid synthesis in familial hypercholesterolemia. *Atherosclerosis*.

[20] Angelin B, Carlson LA (1986). Bile acids and plasma high density lipoproteins: biliary lipid metabolism in fish eye disease. *European Journal of Clinical Investigation*.

[21] Yamori Y, Murakami S, Ikeda K, Nara Y (2004). Fish and lifestyle-related disease prevention: experimental and epidemiological evidence for anti-atherogenic potential of taurine. *Clinical and Experimental Pharmacology & Physiology*.

[22] Morozova S, Suc-Royer I, Auwerx J (2005). Cholesterol metabolism modulators in future drug therapy for atherosclerosis. *Medecine/Sciences*.

[23] Poorman JA, Buck RA, Smith SA, Overturf ML, Loose-Mitchell DS (1993). Bile acid excretion and cholesterol 7*α*-hydroxylase expression in hypercholesterolemia-resistant rabbits. *Journal of Lipid Research*.

[24] Princen HMG, Post SM, Twisk J (1997). Regulation of bile acid biosynthesis. *Current Pharmaceutical Design*.

